# Arsenic Exposure and Glucose Intolerance/Insulin Resistance in Estrogen-Deficient Female Mice

**DOI:** 10.1289/ehp.1408663

**Published:** 2015-04-10

**Authors:** Chun-Fa Huang, Ching-Yao Yang, Ding-Cheng Chan, Ching-Chia Wang, Kuo-How Huang, Chin-Ching Wu, Keh-Sung Tsai, Rong-Sen Yang, Shing-Hwa Liu

**Affiliations:** 1 School of Chinese Medicine, College of Chinese Medicine, China Medical University, Taichung, Taiwan; 2 Department of Surgery,; 3 Department of Geriatrics and Gerontology,; 4 Department of Pediatrics, and; 5 Department of Urology, College of Medicine and Hospital, National Taiwan University, Taipei, Taiwan; 6 Department of Public Health, China Medical University, Taichung, Taiwan; 7 Department of Laboratory Medicine, and; 8 Department of Orthopaedics, College of Medicine and Hospital, National Taiwan University, Taipei, Taiwan; 9 Department of Medical Research, China Medical University Hospital, China Medical University, Taichung, Taiwan; 10 Institute of Toxicology, College of Medicine, National Taiwan University, Taipei, Taiwan

## Abstract

**Background:**

Epidemiological studies have reported that the prevalence of diabetes in women > 40 years of age, especially those in the postmenopausal phase, was higher than in men in areas with high levels of arsenic in drinking water. The detailed effect of arsenic on glucose metabolism/homeostasis in the postmenopausal condition is still unclear.

**Objectives:**

We investigated the effects of arsenic at doses relevant to human exposure from drinking water on blood glucose regulation in estrogen-deficient female mice.

**Methods:**

Adult female mice who underwent ovariectomy or sham surgery were exposed to drinking water contaminated with arsenic trioxide (0.05 or 0.5 ppm) in the presence or absence of 17β-estradiol supplementation for 2–6 weeks. Assays related to glucose metabolism were performed.

**Results:**

Exposure of sham mice to arsenic significantly increased blood glucose, decreased plasma insulin, and impaired glucose tolerance, but did not induce insulin resistance. Blood glucose and insulin were higher, and glucose intolerance, insulin intolerance, and insulin resistance were increased in arsenic-treated ovariectomized mice compared with arsenic-treated sham mice. Furthermore, liver phosphoenolpyruvate carboxykinase (*PEPCK*) mRNA expression was increased and liver glycogen content was decreased in arsenic-treated ovariectomized mice compared with arsenic-treated sham mice. Glucose-stimulated insulin secretion in islets isolated from arsenic-treated ovariectomized mice was also significantly decreased. Arsenic treatment significantly decreased plasma adiponectin levels in sham and ovariectomized mice. Altered glucose metabolism/homeostasis in arsenic-treated ovariectomized mice was reversed by 17β-estradiol supplementation.

**Conclusions:**

Our findings suggest that estrogen deficiency plays an important role in arsenic-altered glucose metabolism/homeostasis in females.

**Citation:**

Huang CF, Yang CY, Chan DC, Wang CC, Huang KH, Wu CC, Tsai KS, Yang RS, Liu SH. 2015. Arsenic exposure and glucose intolerance/insulin resistance in estrogen-deficient female mice. Environ Health Perspect 123:1138–1144; http://dx.doi.org/10.1289/ehp.1408663

## Introduction

Arsenic is a naturally occurring toxic metalloid. Levels of naturally occurring arsenic in groundwater above the drinking water standard of 10 μg/L are not uncommon. Several studies in Taiwan noted that a dose–response relationship between accumulative arsenic exposure and prevalence of diabetes mellitus existed in the villages along the southwestern coast of Taiwan exposed to arsenic-contaminated drinking water [0.1–15 ppm-year (Lai et al. 1994); > 15 ppm-year (Tseng et al. 2002)]. Similar results were also reported in the United States, Iran, and Bangladesh (Mahram et al. 2013; Rahman et al. 1998). Systematic reviews of the literature have suggested that there is an association between arsenic exposure and diabetes (James et al. 2013; Kim et al. 2013; Maull et al. 2012; Navas-Acien et al. 2006; Wang et al. 2014). The NHANES (National Health and Nutrition Examination Survey) 2003–2004 results indicated that exposure to low concentrations of inorganic arsenic in drinking water may play a role in diabetes prevalence (Navas-Acien et al. 2008). Gribble et al. (2012) reported an association between arsenic and diabetes in participants with uncontrolled diabetes (HbA1c ≥ 8%) but not in participants with controlled diabetes. Thus, the detailed effects and mechanisms of arsenic-induced diabetes mellitus still remain to be clarified.

Preclinical studies have indicated that estrogen plays a role in sustaining insulin and glucose homeostasis, which underlies a healthy metabolic profile (reviewed by Rettberg et al. 2014). Ding et al. (2007) suggested that high estrogen and testosterone levels in postmenopausal women are related to an increased risk of developing type 2 diabetes. However, a prospective cohort study found that menopausal hormone therapy was capable of reducing the incidence of new-onset diabetes in women (de Lauzon-Guillain et al. 2009). Therefore, the association of estrogen levels and diabetes mellitus after menopause is complex. In related studies, Chiu et al. (2006) suggested that an association between arsenic exposure and diabetes might be causal for women but not men. An epidemiological study also observed that the prevalence of diabetes in women > 40 years of age, especially those in the postmenopausal stage with low or deficient estrogen levels, was higher than that of men in areas with high levels of arsenic in drinking water (Lai et al. 1994). However, the precise actions of arsenic on diabetes development and the role of estrogen in arsenic-related diabetes remain to be clarified.

In highly contaminated areas in Taiwan, Chen et al. (1994) reported that arsenic levels in the drinking well or underground water were 671 ± 149 (470–897) ppb (mean ± SD and range) and trivalent arsenite was the predominant arsenic species. Arsenic trioxide (As_2_O_3_), a trivalent arsenic compound, is known to be released into the air and water through natural and industrial processes. It is the most commonly produced form of arsenic. As_2_O_3_ can form arsenite in alkaline solution (Eisler 1988; O’Day 2006).

In the present study, we hypothesized that estrogen deficiency is an important factor for arsenic-impaired glucose metabolism/homeostasis in females. We investigated the effects of inorganic arsenic at doses relevant to human exposure from drinking water on blood glucose and insulin, glucose tolerance, and insulin resistance on estrogen-deficient female mice in the presence or absence of estrogen supplementation.

## Materials and Methods

*Animals.* A total of 144 female ICR mice (4 weeks of age, 18.9–22.4 g) were provided by the Animal Center of the College of Medicine, National Taiwan University. The Animal Research Committee of the College of Medicine, National Taiwan University, approved the study, which was conducted in accordance with the guidelines for the care and use of laboratory animals. The animals were treated humanely and with the aim of alleviating suffering. Five mice were housed in each standard rat microisolator cage on aspen chip bedding in an animal room maintained at a constant temperature of 22 ± 2°C with 12-hr light and dark cycles. They were provided with standard chow diet (LabDiet #5053; 5% fat) and water *ad libitum*. All mice were housed for a 1-week acclimation period.

Three weeks after ovariectomy (OVX) or sham surgery, 7-week-old mice were randomly assigned to one of 12 groups and treated with arsenic (As_2_O_3_; Sigma-Aldrich; 0, 0.05, or 0.5 ppm in drinking water) with or without 17β-estradiol (E_2;_ 10 μg/kg/day; subcutaneous injection) for 2–6 weeks. Treatment groups were as follows: sham control and OVX control [double distilled (D.D.) water], sham + arsenic (0.05 ppm), sham + arsenic (0.5 ppm), OVX + arsenic (0.05 ppm), OVX + arsenic (0.5 ppm), sham + E_2_, OVX + E_2_, sham + arsenic (0.05 ppm) + E_2_, sham + arsenic (0.5 ppm) + E_2_, OVX + arsenic (0.05 ppm) + E_2_, and sham + arsenic (0.5 ppm) + E_2_. Each group contained 12 mice. As_2_O_3_ was completely dissolved in alkaline solution and diluted with distilled water to prepare arsenic solutions (pH, 7.2–7.4). To prevent oxidation of As_2_O_3_, the arsenic drinking water was freshly prepared every 3 days.

At each time point (2, 4, and 6 weeks; *n* = 12 mice/group), mice were weighed, food and water consumption were measured, blood samples were collected from the orbital sinus, and levels of glucose and insulin were assayed. In addition, at week 6, blood samples were collected from the tail vein for the oral glucose tolerance test (OGTT; 6 mice/group) and the insulin tolerance test (ITT; 6 mice/group). Finally, after the OGTT and ITT, all animals were sacrificed by decapitation under pentobarbital anesthesia (50 mg/kg, intraperitoneal injection) in the animal housing quarters, and whole blood samples (100 μL) were collected and centrifuged at 3,000 × *g* at 4°C for 10 min to obtain plasma samples. Estradiol and adiponectin levels were assayed immediately using assay kits [estradiol kit (Cayman Chemical Company); adiponectin kit (Assaypro)]. The uterus was removed and weighed, the pancreas was removed for further isolation of islets, and the liver was removed and stored at –80°C.

*Blood glucose and plasma insulin analysis.* Blood samples (50 μL) were collected after 16 hr of fasting using an orbital sinus puncture technique under anesthesia (isoflurane) (Parasuraman et al. 2010; Riley 1960). After removal of the collecting tube, bleeding was stopped by applying gentle pressure to the puncture site using sterile cotton. Blood glucose levels were determined using an Antsense III glucose analyzer (Horiba Industry). Quality control ranges for glucose analysis were 32–56 mg/dL (control-low), 131–160 mg/dL (control-medium), and 396–594 mg/dL (control-high). To measure plasma insulin, whole blood samples were collected from the orbital sinus (50 μL) and centrifuged at 3,000 × *g* at 4°C for 10 min to obtain plasma samples. Aliquots of plasma samples were subjected to insulin antiserum immunoassay (Mercodia, Uppsala, Sweden). Quality control ranges for the insulin analysis were 29.928–45.936 pmol/L (control-low), 102.138–156.426 pmol/L (control-medium), and 579.768–888.096 pmol/L (control-high).

*Insulin resistance index.* Blood samples of 12 mice per group were collected after fasting overnight. To estimate the insulin resistance, HOMA-IR (homeostatic model assessment of insulin resistance) was calculated from the fasting concentrations of insulin and glucose using the following formula: HOMA-IR = fasting serum insulin (mU/L)/[22.5e^–ln fasting plasma glucose (mmol/L)^].

*Oral glucose tolerance test (OGTT).* After exposure to arsenic in drinking water for 6 weeks, 6 mice/group were fasted overnight and received a glucose challenge by oral gavage (1 g/kg body weight). Blood samples were collected before and 15, 45, 75, and 105 min after delivery of the glucose load. Glucose levels in blood from the tail vein (30 μL) were determined using an Antsense-III glucose analyzer (Horiba).

*Insulin tolerance test (ITT).* After exposure to arsenic in drinking water for 6 weeks, 6 mice/group were fasted overnight and received insulin (0.75 U/kg; Actrapid) by intraperitoneal injection. Glucose levels were determined in tail blood (30 μL) at 0, 15, 30, and 60 min after insulin injection.

*Isolated mouse islets.* Mouse islets were isolated as described previously (Chen et al. 2006). Briefly, collagenase (2 mg/mL) in Hank’s solution containing 25 mM HEPES was infused into the main bile duct. The whole pancreas was collected and digested at 37°C. After separation on a Ficoll gradient, the islets were further purified by handpicking four times to eliminate any remaining exocrine tissue. The number of islets was counted by dithizone staining and expressed as the number of islets equivalent to 150 μm in diameter; the islet equivalent quality (1 IEQ = 150 μm) was then calculated. The islets 75–150 μm in diameter were cultured for 24 hr in CMRL1066 medium containing 5.5 mM glucose, 10% fetal bovine serum, 100 U/mL penicillin-streptomycin, 2 mM l-glutamate, 25 mM HEPES, and ITS Premix (5 mg insulin, 5 mg transferrin, and 5 μg selenous acid/L).

*Glucose-stimulated insulin secretion.* The cultured islets were transferred to Krebs Ringer buffer solution (5.5 mM glucose) and then incubated with 20 mM glucose for 1 hr in a 95% air/5% CO_2_ mixture at 37°C. Aliquots of experimental solutions were subjected to insulin antiserum immunoassay (Mercodia) to measure insulin levels.

*Real-time/quantitative polymerase chain reaction (PCR) analysis.* Total RNA was isolated from mouse liver with Trizol reagent (Invitrogen). The relative mRNA expression for phosphoenolpyruvate carboxykinase (*PEPCK*) was determined as previously described (Hsu et al. 2013). Briefly, total RNA (0.5–1.0 μg) was used for the reverse transcription of RNA to cDNA using AMV reverse transcriptase (Promega). cDNA (2 μL) was tested with real-time Sybr Green PCR reagent (Invitrogen) with specific primers (forward/reverse: *PEPCK*: GAGT​GCCC​ATCG​AAGG​​CAT/CCAG​TGCG​CCAG​GTAC​TTG; β-actin: GCCC​TAGA​CTTC​​GAGC/CTTT​ACGG​ATGT​CAAC​GT). The amplification was performed using an ABI StepOnePlus sequence detection system (Applied Biosystems) and analyzed using StepOne software (version 2.1; Applied Biosystems).

*Glycogen assay.* Liver tissue (10 mg) was homogenized and centrifuged at 12,000 rpm for 5 min. The supernatant was collected, and glycogen contents were detected with a glycogen assay kit (BioVision).

*Determination of arsenic content.* Arsenic concentrations in blood and urine were measured by dynamic reaction cell inductively coupled plasma–mass spectrometry (Elan DRC ICP-MS; Perkin Elmer) according to the U.S. Centers for Disease Control and Prevention laboratory methods for metals in whole blood (DLS Method code-CTL-TMS-3.01; CDC 2012). Indium was used as an internal standard, and the limit of detection was 0.24 μg As/L.

*Statistical analysis.* Data are presented as mean ± SEM. Data were analyzed for statistical significance using one-way analysis of variance followed by Holm–Sidak post hoc analysis to test for differences between groups. A *p* ≤ 0.05 was considered statistically significant. Statistical analyses were performed using SPSS 16.0 software (SPSS).

## Results

**Figure 1:**
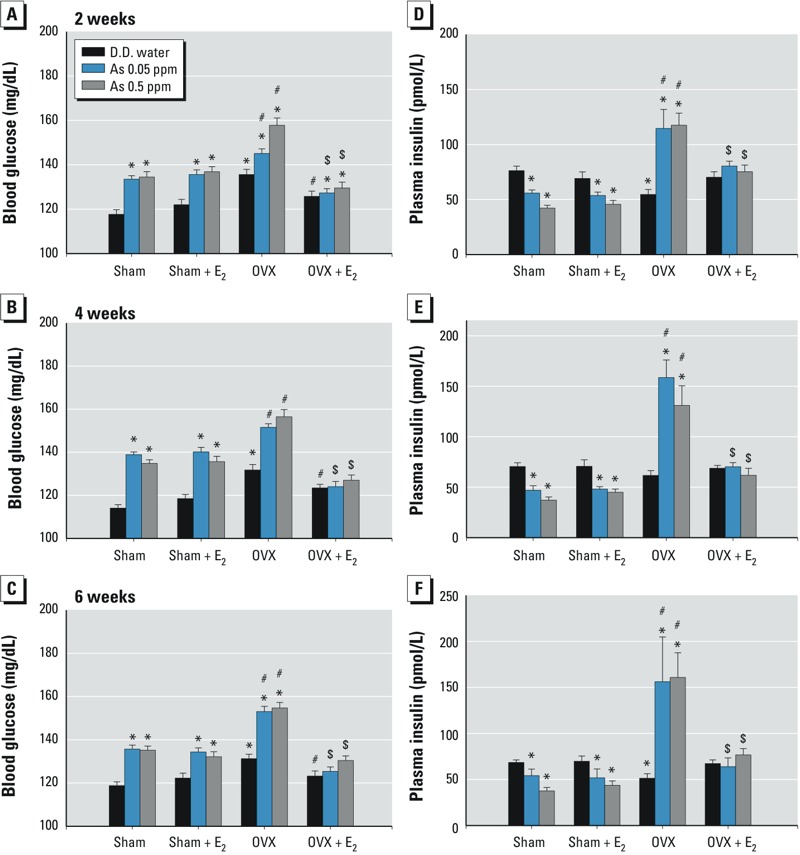
Effects of arsenic on the blood glucose and plasma insulin levels in female mice with or without ovariectomy. Mice were treated with D.D. water or with As_2_O_3_ (0.05 or 0.5 ppm] in drinking water in the presence or absence of 17β-estradiol (E_2_; 10 μg/kg) for 2–6 weeks. (*A–C*) Blood glucose levels. (*D–F*) Plasma insulin levels. Data are mean ± SEM (*n *= 12/group). *p < 0.05 vs. sham D.D. water. ^#^*p *< 0.05 vs. OVX D.D. water. ^$^*p *< 0.05 vs. OVX with As_2_O_3_.

*Effects of arsenic on blood glucose and insulin in estrogen-deficient mice.* Body weights and weight gains were significantly increased and uterine weights were significantly decreased in OVX mice compared with sham mice, but they were reversed by E_2_ (Table 1). Exposure to arsenic (0.05 and 0.5 ppm) had no effect on uterine weights or body weight gain. Arsenic did not affect food and water consumption in any of the arsenic-treated groups (data not shown), which was consistent with the report of Ambrosio et al. (2014). Arsenic significantly increased the blood glucose levels in sham mice, and blood glucose levels were higher in OVX mice than those in sham mice exposed to arsenic, which could be reversed by E_2_ (Figure 1A–C). Arsenic treatment significantly decreased the plasma insulin levels in sham mice, whereas arsenic significantly enhanced the plasma insulin levels in OVX mice, which could be reversed by E_2_ (Figure 1D–F).

**Table 1:** Effects of arsenic (0.05 or 0.5 ppm) on uterine and body weights at 6 weeks in female mice with or without ovariectomy.

Treatment	Uterine weight		Body weight/gain (g)
	mg	Fold increase	
Sham	181.1 ± 15.8	—	32.4 ± 0.3/1.61 ± 0.28
As 0.05	215.2 ± 17.0	1.19	33.3 ± 0.4/1.58 ± 0.43
As 0.5	182.6 ± 16.4	1.01	33.0 ± 1.3/1.38 ± 0.21
Sham + E_2_	217.0 ± 9.2	1.20	35.3 ± 0.8/2.02 ± 0.23
As 0.05 + E_2_	213.0 ± 11.5	1.18	35.4 ± 0.6/2.18 ± 0.24
As 0.5 + E_2_	204.7 ± 14.3	1.13	35.3 ± 0.7/1.95 ± 0.56
OVX	20.9 ± 3.0*	0.12	39.6 ± 1.0/6.45 ± 1.10*
OVX + As 0.05	19.7 ± 1.9*	0.11	37.2 ± 0.8/6.24 ± 1.40*
OVX + As 0.5	19.8 ± 2.3*	0.11	38.5 ± 1.3/6.65 ± 1.43*
OVX + E_2_	105.6 ± 3.2*^,#^	0.58	33.8 ± 0.5/1.88 ± 0.47^#^
OVX + As 0.05 + E_2_	104.4 ± 5.0*^,#^	0.58	33.8 ± 0.6/1.83 ± 0.46^#^
OVX + As 0.5 + E_2_	96.6 ± 4.3*^,#^	0.53	32.1 ± 0.7/1.81 ± 0.37^#^
Abbreviations: As, As_2_O_3_; E_2_, 17β-estradiol; OVX, ovariectomy. Values are means ± SEM (*n* = 12). *p < 0.05 vs. sham. ^#^*p *< 0.05 vs. OVX.			

**Figure 2:**
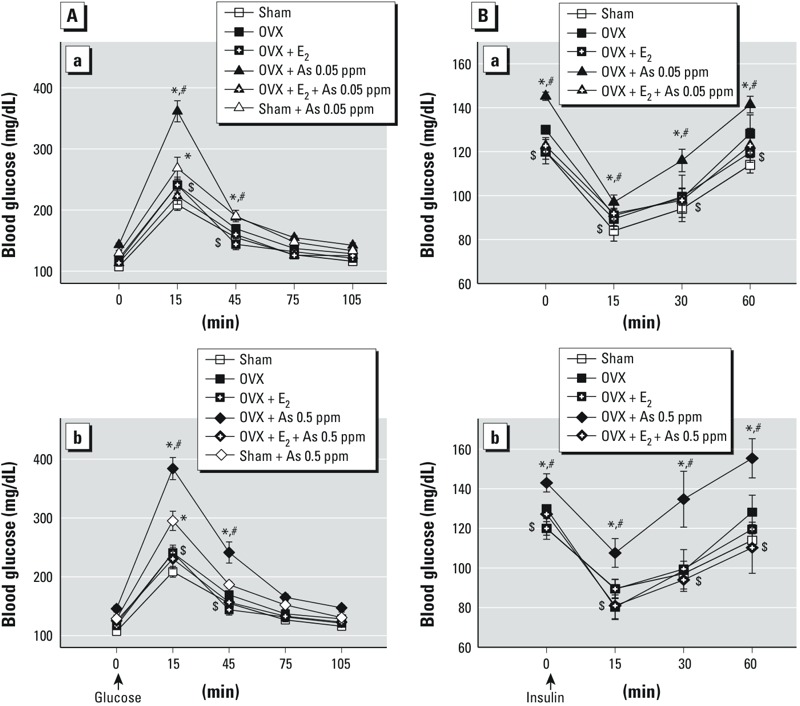
Effects of arsenic on the oral glucose tolerance test (OGTT; *A*) and the insulin tolerance test (ITT; *B*) in female mice with or without ovariectomy. Mice were treated with D.D. water or with As_2_O_3_ (As) 0.05 ppm (a) or 0.5 ppm (b) in drinking water in the presence or absence of 17β-estradiol (E_2_; 10 μg/kg) for 6 weeks. Data are mean ± SEM (*n *= 6/group). *p < 0.05 vs. sham D.D. water. ^#^*p *< 0.05 vs. OVX D.D. water. ^$^*p *< 0.05 vs. OVX + As_2_O_3_.

*Effects of arsenic on glucose and insulin tolerance and insulin resistance in estrogen-deficient mice.* As shown in Figure 2A, arsenic treatment (a, 0.05 ppm; b, 0.5 ppm) for 6 weeks significantly impaired glucose tolerance in sham mice. Arsenic induced greater impairment of glucose tolerance in OVX mice than that in sham mice, which could be reversed by E_2_. Furthermore, arsenic significantly impaired insulin tolerance (Figure 2Ba, 0.05 ppm; 2Bb, 0.5 ppm) and increased HOMA-IR (Table 2) in OVX mice, which could be reversed by E_2_. E_2_ had no effects and did not interact significantly with arsenic. Arsenic did not increase the insulin resistance in sham mice (Table 2).

**Table 2:** Effects of arsenic (0.05 or 0.5 ppm) on HOMA-IR in female mice with or without ovariectomy in the presence or absence of 17β-estradiol (E_2_).

Treatment	2 week	4 week	6 week
Sham control	1.91 ± 0.24	1.77 ± 0.10	1.64 ± 0.16
Sham + As 0.05	2.03 ± 0.11	1.85 ± 0.15	1.66 ± 0.13
Sham + As 0.5	1.87 ± 0.11	1.94 ± 0.31	1.65 ± 0.12
Sham + E_2_	2.15 ± 0.29	2.54 ± 0.27	1.70 ± 0.20
Sham + As 0.05 + E_2_	2.14 ± 0.09	1.77 ± 0.21	1.74 ± 0.13
Sham + As 0.5 + E_2_	2.33 ± 0.22	1.70 ± 0.20	1.78 ± 0.15
OVX control	2.89 ± 0.29	2.56 ± 0.32	2.44 ± 0.26
OVX + As 0.05	4.40 ± 0.86*	6.68 ± 1.00*	4.31 ± 0.28*
OVX + As 0.5	5.65 ± 1.11*	3.54 ± 0.29*	3.20 ± 0.34*
OVX + E_2_	2.35 ± 0.24	2.34 ± 0.40	1.71 ± 0.14
OVX + As 0.05 + E_2_	2.41 ± 0.25^#^	2.97 ± 0.37^#^	2.23 ± 0.16^#^
OVX + As 0.5 + E_2_	2.61 ± 0.20^#^	2.47 ± 0.35^#^	2.10 ± 0.20^#^
Abbreviations: As, As_2_O_3_; E_2_, 17β-estradiol; OVX, ovariectomy. Values are mean ± SEM (*n* = 12/group). *p < 0.05 vs. sham control. ^#^*p *< 0.05 vs. OVX control.			

**Figure 3:**
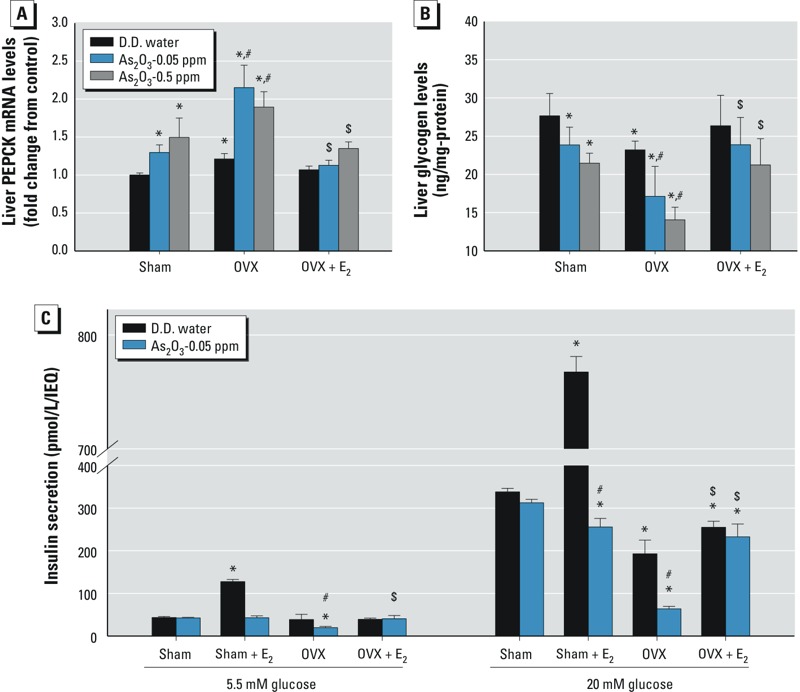
Effects of arsenic on *PEPCK* mRNA expression and glycogen content in liver and on insulin secretion in islets of female mice with or without ovariectomy. Mice were treated with D.D. water or with As_2_O_3_ (0.05 or 0.5 ppm) in drinking water in the presence or absence of 17β-estradiol (E_2_; 10 μg/kg) for 6 weeks. The liver was isolated to analyze *PEPCK* mRNA expression (*A*) and glycogen content (*B*). The pancreatic islets were isolated to analyze glucose-stimulated insulin secretion (*C*). Data are mean ± SEM (*n *= 6/group). *p < 0.05 vs. sham D.D. water. ^#^*p *< 0.05 vs. OVX D.D. water. ^$^*p *< 0.05 vs. OVX with As_2_O_3_.

*Effects of arsenic on liver PEPCK expression, glycogen content, and islet insulin secretion in estrogen-deficient mice.* Arsenic treatment for 6 weeks significantly increased liver *PEPCK* mRNA expression (Figure 3A) and decreased liver glycogen content (Figure 3B) in sham mice. OVX mice had higher liver *PEPCK* mRNA expression and lower liver glycogen content than sham control mice, which could be reversed by E_2_ supplementation.

Glucose-stimulated insulin secretion in islets isolated from arsenic-treated OVX mice was significantly decreased, although there was no significant change in the islets of arsenic-treated sham mice without E_2_ (Figure 3C). In sham control mice, E_2_ markedly increased the 20-mM glucose-stimulated insulin secretion in the islets, which could be significantly inhibited by arsenic treatment. E_2_ significantly reversed the inhibition of insulin secretion in the islets of arsenic-treated OVX mice.

**Figure 4:**
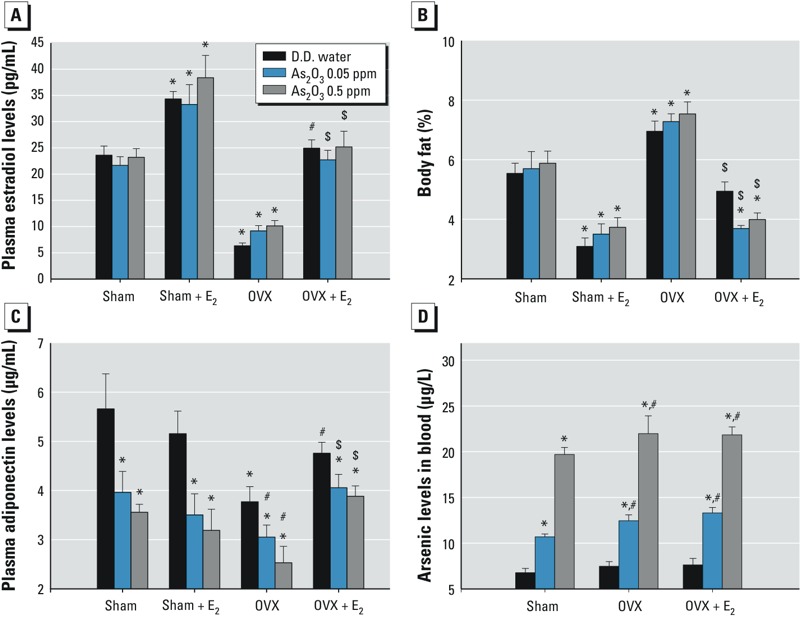
Effects of arsenic on the levels of serum estradiol (*A*), total fat (*B*), serum adiponectin (*C*), and blood arsenic (*D*) in female mice with or without ovariectomy. Mice were treated with D.D. water or with As_2_O_3_ (0.05 or 0.5 ppm) in drinking water in the presence or absence of 17β-estradiol (E_2_; 10 μg/kg) for 6 weeks. Data are mean ± SEM (*n *= 12/group). *p < 0.05 vs. sham D.D. water. ^#^*p* < 0.05 vs. OVX D.D. water. ^$^*p* < 0.05 vs. OVX with As_2_O_3_.

*Effects of arsenic on the levels of estradiol, fats, and adiponectin in estrogen-deficient mice.* In OVX mice, there were lower levels of serum estradiol, higher levels of body fats, and lower levels of plasma adiponectin, all of which could be significantly reversed by E_2_ (Figure 4A–C). Arsenic treatment for 6 weeks did not affect the levels of serum estradiol and body fats, but significantly decreased plasma adiponectin levels in sham and OVX mice. We noted lower levels of plasma adiponectin in arsenic-treated OVX mice than in OVX control mice or in arsenic-treated sham mice (Figure 4C). Moreover, the arsenic content in blood was increased in all arsenic-treated mice with or without OVX in a dose-dependent manner (Figure 4D). The urinary arsenic levels in sham control mice and mice treated with As_2_O_3_ arsenic 0.05 ppm and arsenic 0.5 ppm for 2 weeks were 96.00 ± 10.35, 175.22 ± 13.58, and 713.89 ± 30.71 μg/L, respectively (*p* < 0.05 vs. sham control; *n* = 12 per group).

## Discussion

Recent epidemiological evidence has supported a link between inorganic arsenic exposure and the increased prevalence of diabetes in populations exposed to arsenic in drinking water (Maull et al. 2012). Gribble et al. (2012) reported that urine arsenic was associated with poor diabetes control in a population of American Indians from rural communities in the United States with a high burden of diabetes. An association between increased risk for type 2 diabetes and lifetime exposure to low-level inorganic arsenic (< 100 μg/L) in drinking water was also reported in a case–cohort study (James et al. 2013). Kim et al. (2013) showed that modestly elevated exposure to inorganic arsenic was a predictor of type 2 diabetes in a population of American Indians from Arizona who have a high prevalence of type 2 diabetes. Exposure to high-dose inorganic arsenic in drinking water (25 or 50 ppm) has been shown to synergistically act with high fat diet–induced obesity in impairing glucose tolerance in mice (Paul et al. 2011). Low subtoxic concentrations of arsenite and its methylated trivalent metabolites have also been found to inhibit glucose-stimulated insulin secretion, but not basal insulin secretion, in isolated mouse pancreatic islets (Douillet et al. 2013). In the present study, we demonstrated that exposure to inorganic arsenic at doses relevant to human exposure from drinking water increased blood glucose levels, impaired glucose tolerance, and decreased plasma insulin levels, but did not cause insulin resistance in sham control female mice. However, OVX-induced estrogen deficiency exacerbated hyperglycemia and glucose intolerance, increased plasma insulin levels, and induced insulin intolerance and insulin resistance in female mice exposed to arsenic. The characterization of arsenic-related diabetes observed in epidemiological studies is similar to type 2 diabetes (Navas-Acien et al. 2006). Exposure to highly contaminated drinking water (arsenic levels > 100 ppb) has been shown to be associated with an increased risk of type 2 diabetes in Taiwan and Bangladesh (Lai et al. 1994; Rahman et al. 1998). Likewise, our results indicate that estrogen-deficient female mice exposed to arsenic experience an exacerbated diabetic state that resembles type 2 diabetes.

The present work also demonstrated that E_2_ supplementation effectively reversed the exacerbated hyperglycemia/glucose intolerance and induced insulin resistance in ovariectomized female mice. Estrogen is known to exert its effects mainly through the estrogen receptor (ER), and estrogen/ER signaling is a well-known regulator of metabolic and glucose-sensing tissues, including pancreatic β cells, liver, adipocyte, and skeletal muscle (Faulds et al. 2012). Le May et al. (2006) reported that E_2_ acting through ER-α prevented pancreatic β cell apoptosis and diabetes in an insulin-deficient diabetic mouse model. Hormone replacement therapy has also been found to improve hyperglycemic control and to decrease the incidence of type 2 diabetes in postmenopausal women (Khoo and Perera 2005). In contrast, Lai et al. (1994) reported that women had a higher prevalence of diabetes mellitus than men after 40 years of age, especially in the postmenopausal phase (age > 50 years). Therefore, our results in an estrogen-deficient female mouse model and the findings of previous epidemiological studies support the possibility that estrogen deficiency is an important risk factor in arsenic-induced glucose metabolism/homeostasis damage.

Hepatic glycogen plays a major role in the maintenance of blood glucose homeostasis. Breakdown of glycogen in hepatocytes can release glucose into the bloodstream. Glycogenolysis is known to contribute to excessive glucose production in type 2 diabetic patients. Dysregulation of glucose-6-phosphate by changes in the activity of glucokinase or glucose 6-phosphatase in type 2 diabetes may be a contributing factor in the impaired suppression of glycogenolysis caused by hyperglycemia (Aiston et al. 2003). Basu et al. (2005) observed that hepatic insulin resistance in obese nondiabetic and type 2 diabetic humans could be induced by impairment of the regulation of glycogenolysis as well as gluconeogenesis.

PEPCK is a key rate-controlling enzyme that catalyzes the first committed step in hepatic gluconeogenesis. Friedman et al. (1993) suggested that the lack of *PEPCK* gene expression may cause defective insulin signaling and induce hyperglycemia in diabetic animal models. Arsenic was shown to increase both basal and hormone-induced *PEPCK* gene expression in cultured rat hepatoma H411E cells at non-overtly toxic doses (Hamilton et al. 1998). Inorganic arsenic is capable of impairing glucose-stimulated insulin secretion in cultured pancreatic β cells via the induction of oxidative stress (Fu et al. 2010). In the present study, we found that arsenic treatment increased liver *PEPCK* expression and decreased liver glycogen content in sham mice. Estrogen deficiency signifcantly enhanced the increased liver *PEPCK* expression and decreased liver glycogen content in arsenic-treated mice. Moreover, the glucose-stimulated insulin secretion in islets isolated from arsenic-treated OVX mice was significantly decreased. E_2_ supplementation significantly reversed the increased liver *PEPCK* expression, decreased liver glycogen content, and decreased islet insulin secretion in arsenic-treated OVX mice. These results indicate that estrogen deficiency enhances impairments in the regulations of liver gluconeogenesis and glycogenolysis and islet insulin secretion in arsenic-treated mice.

Adiponectin is an adipokine that is released from adipose tissue and directly sensitizes the body to insulin. Hypoadiponectinemia has been suggested to play an important causal role in obesity-linked insulin resistance, type 2 diabetes, and metabolic syndrome (reviewed by Kadowaki et al. 2006). A meta-analysis has also shown that higher adiponectin levels were associated with a lower risk of type 2 diabetes (Li et al. 2009). Adiponectin can also augment high glucose–induced insulin secretion from pancreatic islet β cells (Gu et al. 2006). In the present study, we found that arsenic treatment significantly decreased plasma adiponectin levels in sham and OVX mice. These results suggest that reduced adiponectin may play a causal role in arsenic-induced impairment of glucose homeostasis. However, the detailed mechanism remains to be clarified.

Orbital sinus puncture is a blood-collection technique recommended by the institutional animal care and utilization committees of many institutions, and many animal studies have used this technique to collect blood samples. For example, 50-μL blood samples for plasma glucose determination were collected from the orbital sinus in alloxan-diabetic mice (Ajabnoor and Tilmisany 1988), and 25-μL blood samples for OGTT were collected from the orbital sinus in *db/db* diabetic mice (Gibbs et al. 1995). Christensen et al. (2009) and Shirasaki et al. (2012) suggested collecting 30- to 50-μL blood samples from the orbital sinus or tail vein. In the present study, 30- to 50-μL blood samples were collected from the orbital sinus or tail vein for blood glucose and insulin analysis in mice.

Overnight fasting, used in the present study, is a very common procedure in pharmacological/toxicological studies. Overnight fasting is also a common procedure in experiments of glucose metabolism or OGTT (e.g., Ajabnoor and Tilmisany 1988; Gibbs et al. 1995). Typically, mice are fasted for either 14–18 hr (overnight fast) or 5–6 hr (morning fast) in metabolic experiments (Agouni et al. 2010; Ayala et al. 2010). Matsuzawa and Sakazume (1994) found that plasma glucose levels were unchanged in male F344 rats and male beagle dogs after 16 hr of fasting, although there were changes in some plasma enzymes or lipids. Moreover, prolonged fasting has been found to enhance, but not impair, insulin-stimulated glucose utilization in normal mice, which is different than the situation in humans (Ayala et al. 2006). It has been suggested that fasting 5–6 hr is sufficient to assess physiological responses to glucose metabolism in mice (Agouni et al. 2010). Ayala et al. (2010) suggested that an overnight fast was useful for studies of glucose utilization, and 5–6 hr of fasting is sufficient for assessing insulin action within a more physiological condition.

The biomarkers of arsenic exposure can be assessed from urine, blood, hair, or nails (NRC 1999; WHO 2001), although urinary arsenic is considered to be the most common biomarker of inorganic arsenic exposure (Hughes 2006). Because absorbed arsenic is cleared from blood within a few hours, blood arsenic analysis is suited for recent high-dose exposure (NRC 1999). In humans, absorbed arsenic is primarily excreted in urine, with an approximately 4-day half-life (NRC 1999; WHO 2001). The present results showed that both blood and urinary arsenic levels were significantly increased in arsenic-treated mice.

Our data revealed no significant difference over a 10-fold range of arsenic doses under the treatment conditions. To assess this, we measured the arsenic contents in blood of exposed mice. The blood arsenic contents in sham mice treated with 0.05 and 0.5 ppm arsenic were 10.69 ± 0.32 and 19.70 ± 0.76 ppb, respectively. There was no significant change in blood arsenic content in arsenic-treated OVX mice. Therefore, there was only about a 2-fold range of blood arsenic contents in mice treated with 0.05 and 0.5 ppm arsenic, suggesting no obvious dose-dependent effect of arsenic under the treatment conditions. Moreover, inorganic arsenic is known to be metabolized to methylated compounds (predominantly methylrsonate and dimethylarsinate), which can be largely cleared through the urine in humans (Navas-Acien and Guallar 2008). Sex and age differences in arsenic metabolism have also been reported in a population in Bangladesh, where water arsenic concentrations are high (Lindberg et al. 2008). The changes in arsenic metabolism in arsenic-treated estrogen-deficient mice remain to be fully investigated.

## Conclusions

In this study, we examined the role of estrogen in arsenic-related diabetes in female mice. We demonstrated that OVX-induced estrogen deficiency exacerbated hyperglycemia and glucose intolerance, increased plasma insulin levels, and induced insulin intolerance and insulin resistance in female mice exposed to inorganic arsenic in drinking water at doses relevant to human exposure. Moreover, we found that the regulation of liver gluconeogenesis and glycogenolysis and islet insulin secretion were markedly impaired by arsenic exposure in estrogen-deficient female mice, which could be effectively reversed by estrogen supplementation. These findings suggest that estrogen deficiency plays an important role in the alteration of glucose metabolism/homeostasis in females during arsenic exposure.
